# Management of No-Reflow With Intracoronary Thrombolysis: A Case Report

**DOI:** 10.7759/cureus.105951

**Published:** 2026-03-27

**Authors:** Youssef Fihri, Driss Britel, Fatima Ezahra Elktaibi, Aatif Benyass, Zouhair Lakhal

**Affiliations:** 1 Cardiac Intensive Care Unit, Military Hospital Mohamed V, Rabat, MAR; 2 Cardiac Catheterization Unit, Military Hospital Mohamed V, Rabat, MAR; 3 Cardiology Center, Military Hospital Mohamed V, Rabat, MAR; 4 Cardiac Intensive Care Unit, Cardiac Catheterization Unit, Military Hospital Mohamed V, Rabat, MAR

**Keywords:** acute stent thrombosis, catheter-directed thrombolysis, no-reflow, right coronary artery (rca), tenecteplase (tnk)

## Abstract

No-reflow is a recurring phenomenon during coronary angioplasty that consists of the failure to reestablish blood flow after clearing an occluded coronary artery. Several treatments have been proposed for the management of no-reflow, including intracoronary thrombolysis, which has emerged in recent years as one of the means of managing this phenomenon. We report the case of a 68-year-old female patient with a high cardiovascular risk, admitted for the management of non-ST-elevation acute coronary syndrome, who presented a no-reflow phenomenon after angioplasty of the right coronary artery. After the failure of the various means available to reopen the artery, a 30 mg dose of tenecteplase was administered intracoronary with restoration of Thrombolysis in Myocardial Infarction III flow in the angiographic control and good clinical and rhythmic evolution. Fibrinolytic agents remain an effective means in the treatment of acute coronary syndromes with ST-segment elevation. Its use in intracoronary therapy has demonstrated its benefit in restoring flow and improving microcirculation while maintaining a satisfactory safety profile. The use of these agents in the treatment of no-reflow remains an alternative to the usual treatment, such as adenosine and verapamil, and mechanical means, such as thrombo-aspiration, allowing dissolution of the thrombus and reestablishment of the flow. The management of no-reflow phenomena remains difficult, given the varied pathophysiology of this phenomenon and the therapeutic arsenal available for its management. However, intracoronary thrombolysis for the management of no-reflow remains an attractive and interesting alternative with results that remain acceptable, but which need to be approved by studies on larger samples with more details on the dose and the administration methodology.

## Introduction

Since the confirmation of the role of thrombus in the pathophysiology of ST-elevation myocardial infarction (STEMI), intracoronary thrombolysis has been the first means to show its effectiveness in myocardial reperfusion [1]. In fact, fibrinolysis has long been the primary therapy for treating acute coronary syndrome with ST-segment elevation. Given the evolution of various coronary angioplasty techniques over the years, its use has declined exponentially, particularly with the advent of primary percutaneous coronary intervention (PPCI) as a reliable and less risky alternative. No-reflow phenomenon consists of a failure to restore optimal coronary flow after the opening of an occluded coronary artery [2]. This can occur in the context of an acute coronary syndrome (this can complicate 60% of STEMI cases) or in a stable patient [2].

The importance of intracoronary thrombolysis has begun to resurface, particularly in certain cases of early stent thrombosis and no-reflow, making the use of fibrinolytic agents in combination with PCI a very interesting alternative to address these conditions.

We report the case of a 68-year-old female patient with a high cardiovascular risk, admitted for the management of non-ST-elevation acute coronary syndrome, who presented a no-reflow phenomenon after angioplasty of the right coronary artery.

## Case presentation

We report the case of a 68-year-old female patient, mother of four children, known to be hypertensive under treatment, and a diabetic for 10 years under insulin, which was poorly controlled with the last HbA1c at 13%, with dyslipidemia under statins. The patient was known to have coronary artery disease since 2015. At the time, the patient presented with a non-ST-segment elevation acute coronary syndrome stented on the proximal left anterior descending (LAD) artery. In 2021, the patient presented with an in-stent restenosis with the placement of three stents covering the entire LAD artery with good evolution. The patient was admitted during her current hospitalization for management of constrictive retrosternal chest pain, prolonged related to a non-ST-elevation acute coronary syndrome. The clinical examination revealed a conscious, eupneic patient without pain, with a blood pressure of 116/64 mmHg and a heart rate of 86 beats/minute. The cardiovascular examination was normal, as was the rest of the clinical examination.

**Figure 1:**
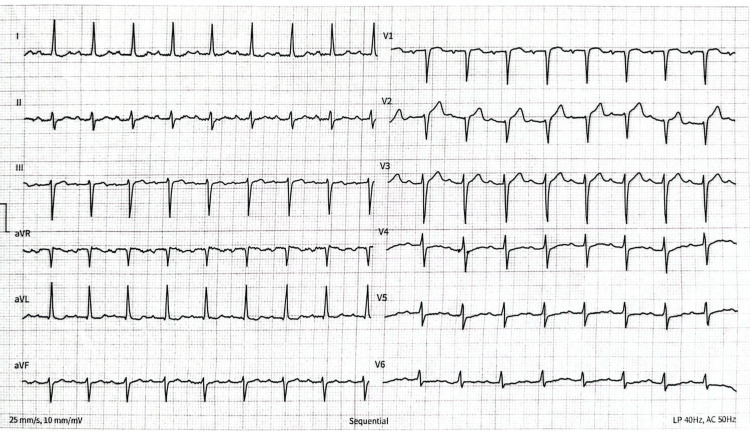
ECG before admission to the catheterization room.

The initial ECG showed a regular sinus rhythm with a heart rate of 105 beats/minute in the presence of a first-degree atrioventricular block with a PR interval of 240 ms, with the presence of an anteroseptal R wave abrasion and an ST-segment elevation in AVr (Figure 1).

The biological assessment showed a hemoglobin of 13.7 g/dL, leukocyte count of 7,500/mm³, platelet count of 230,000/mm³, potassium of 4.3 mmol/L, creatinine of 7 mg/L, and a troponin count that went from 22 times the normal level to 39 times (Table 1).

**Table 1:** Biological values observed in the patient at the time of admission.

Biological parameter	Patient value	Reference value
Hemoglobin	13.7 g/dL	13-17 g/dL
Leukocytes	7,500/mm³	4,000–10,000/mm³
Platelets	230,000/mm³	150,000–450,000/mm³
Creatinine	7 mg/L	6–13 mg/L
Troponin	1,344 ng/L to 966 ng/L	2–34 ng/L

Echocardiography demonstrated a non-dilated, non-hypertrophied left ventricle with hypokinesia of the anteroseptal and anterior wall and the inferior wall with an estimated left ventricular ejection fraction of 50%. The right ventricle was of normal size and function with non-elevated filling pressures and a low probability of pulmonary hypertension.

The patient received 180 mg of ticagrelor and 300 mg of aspirin combined with unfractionated heparin and 80 mg of atorvastatin before admission for coronary angiography.

**Figure 2:**
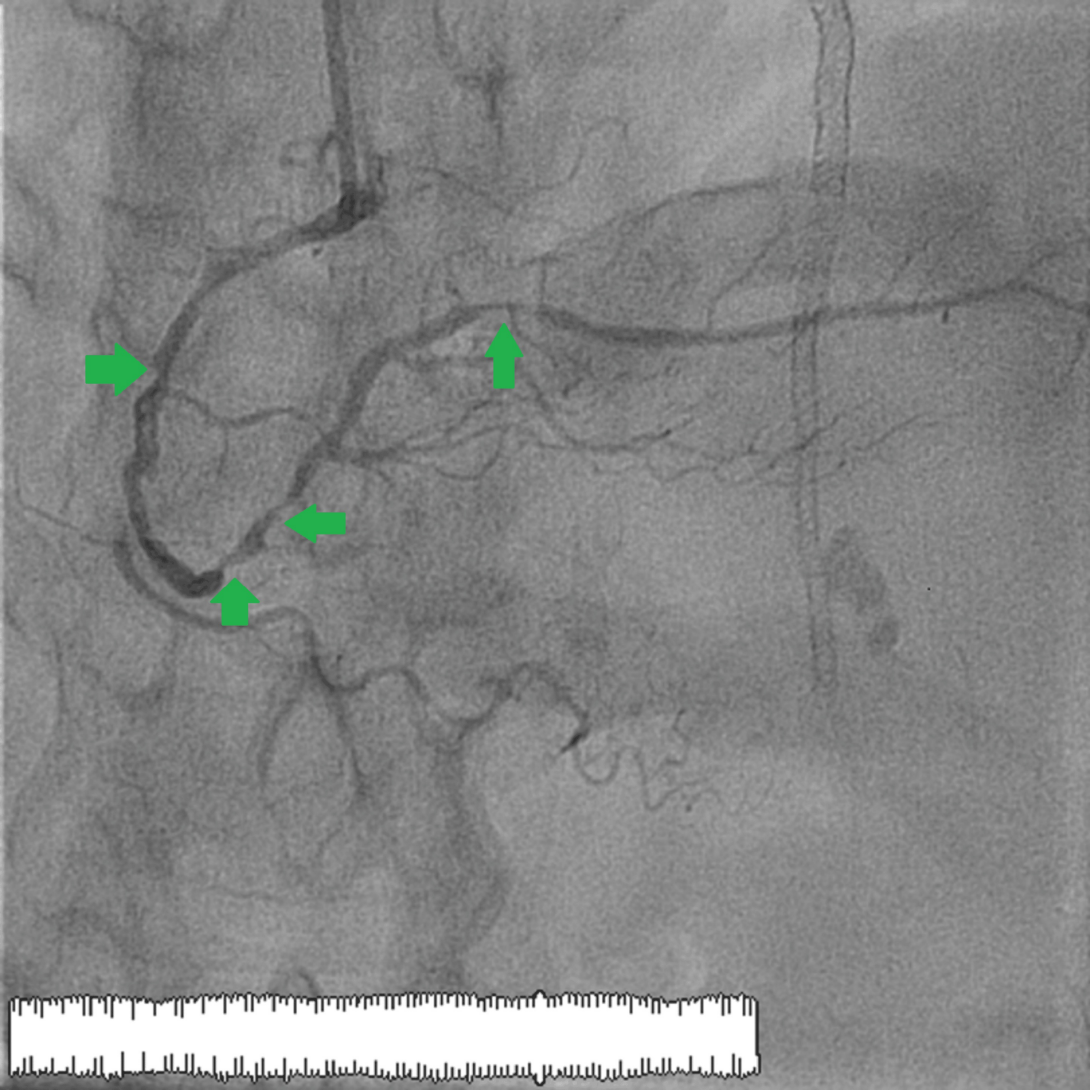
The right coronary artery showing multiple stenoses involving the middle and distal part as well as the middle segment of the posterior retro-ventricular artery. The green arrows show the locations of the different stenoses observed on the right coronary artery and the posterior retro-ventricular artery.

Coronary angiography showed no restenosis in the drug-eluting stents present in the LAD artery, with significant stenosis in the middle circumflex artery and the first marginal artery. The right coronary system was completely diseased with several tight stenoses in the middle and distal right coronary artery and the posterior retro-ventricular artery (Figure 2). The presence of triple trunk lesions likely explained the ST-segment elevation present in the AVr lead.

**Figure 3:**
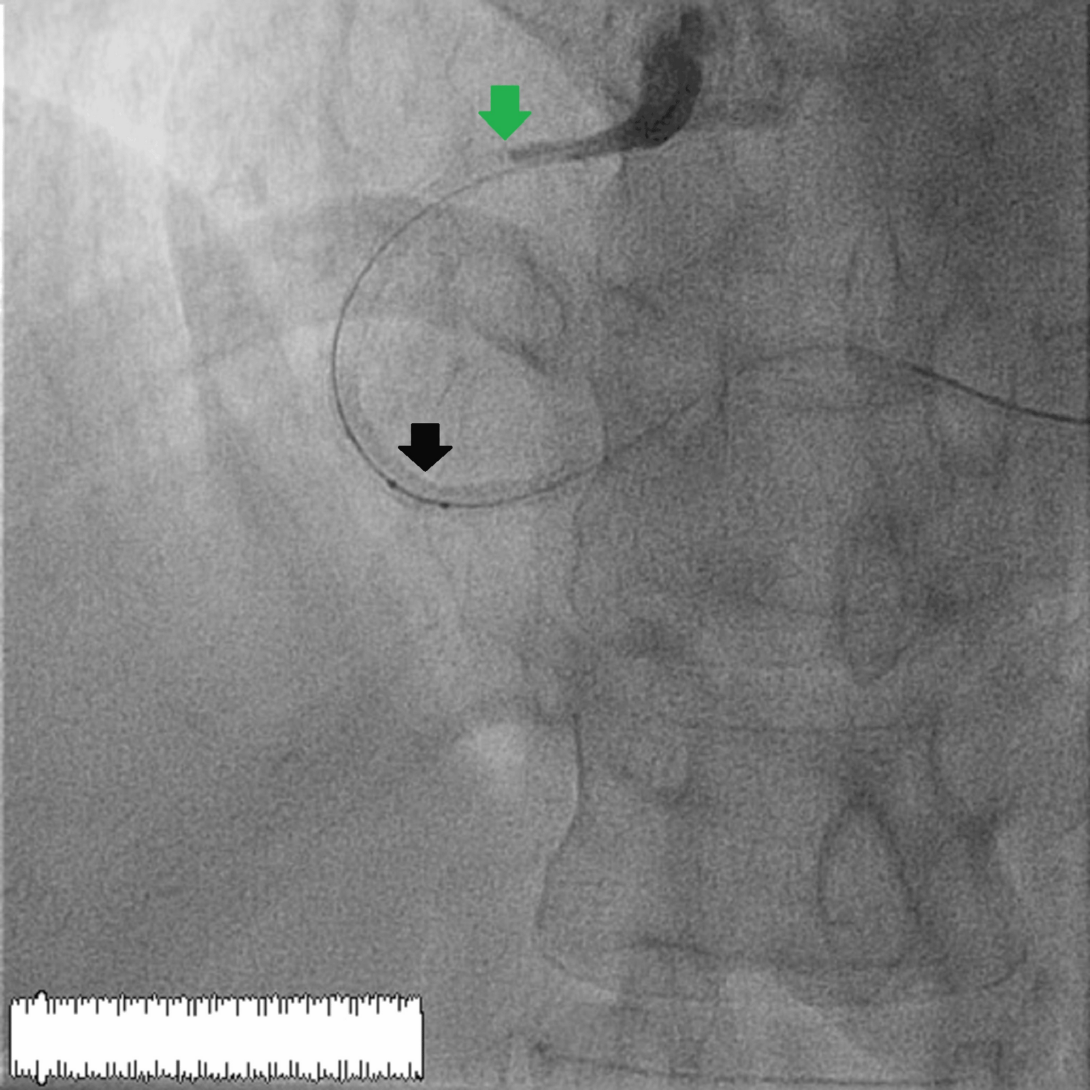
No-reflow phenomenon with Thrombolysis in Myocardial Infarction 0 flow from the proximal part of the right coronary artery with installation of a non-compliant balloon. The green arrow shows the beginning of occlusion at the stent entrance. The black arrow shows the non-compliant balloon used before inflation.

We decided to perform angioplasty of the right coronary artery and the posterior retro-ventricular artery. We started by pre-dilating the different lesions with a 2 × 12 mm semi-compliant balloon, then we placed a 2.5 × 13 mm drug-eluting stent at the posterior retro-ventricular artery with good control. Afterwards, we proceeded to place a 2.5 × 26 mm drug-eluting stent in the distal right coronary artery with satisfactory control. And as a final step, we chose to place a 2.5 × 35 mm drug-eluting stent in the middle right coronary artery. The final angiographic control demonstrated early thrombosis of the different stents with a Thrombolysis in Myocardial Infarction (TIMI) 0 flow and the presence of an intraluminal thrombus with the onset of intense chest pain and ST-segment elevation in the inferior territory, with the occurrence of a complete atrioventricular block with a heart rate of 35 beats/minute. We started by setting up an external temporary pacing for the stabilization of the rhythm, and then we started to inject nitrates intracoronary, followed by the realization of several balloon inflations with a non-compliant balloon of different sizes and diameters (Figure 3).

Despite the multitude of balloon inflations with different diameters, the no-reflow persisted. The decision to perform thrombo-aspiration was made. However, the latter did not bring back any debris with a control showing a persistent TIMI 0 flow. Given the absence of other solutions, it was decided to administer intracoronary fibrinolytics with a dose of 30 mg of tenecteplase.

**Figure 4:**
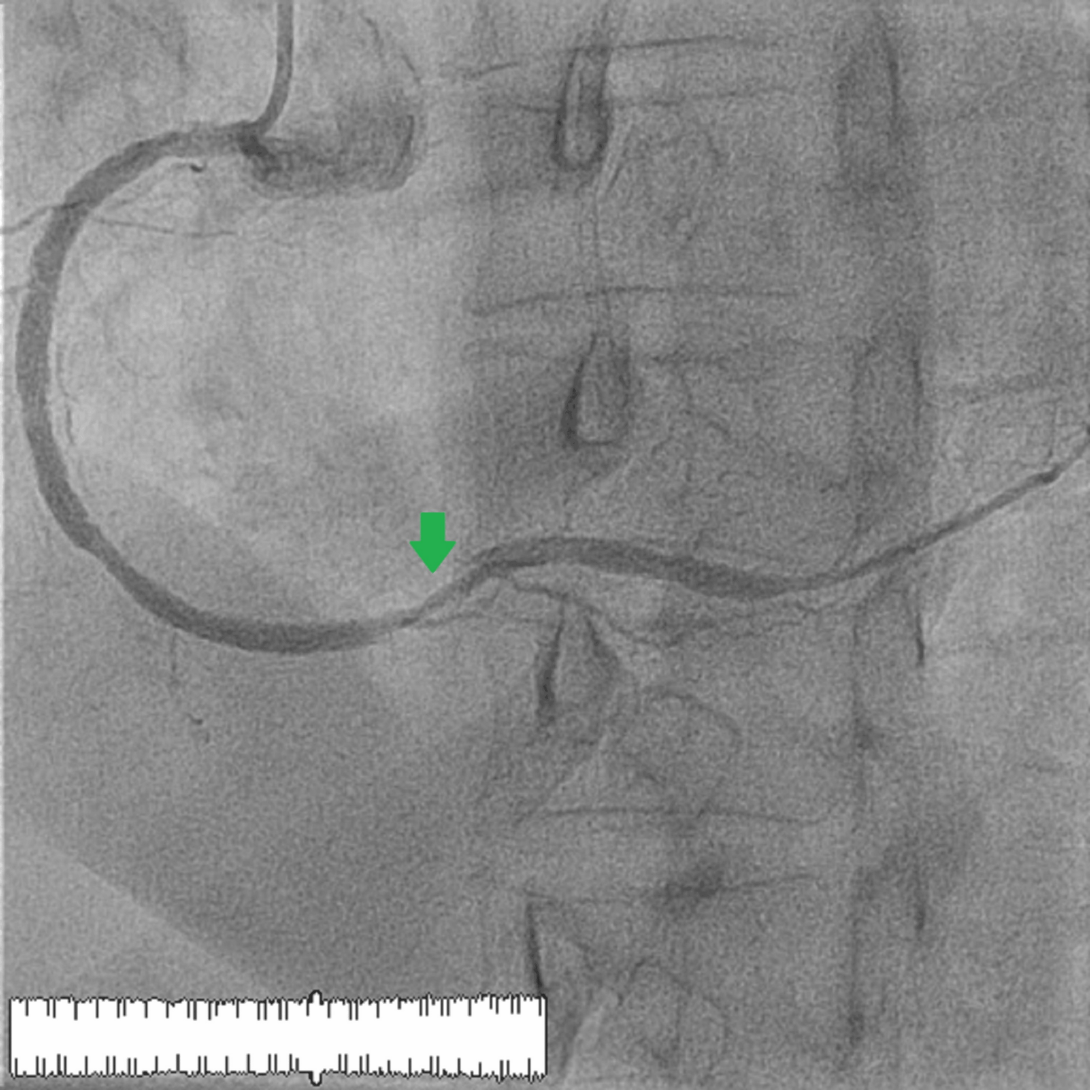
Right anterior oblique incidence at 30 degrees showing the reestablishment of flow with the persistence of a slight thrombus distal to the right coronary artery and the proximal part of the posterior retro-ventricular artery. The green arrow shows the migration of the thrombus toward the distal right coronary artery.

**Figure 5:**
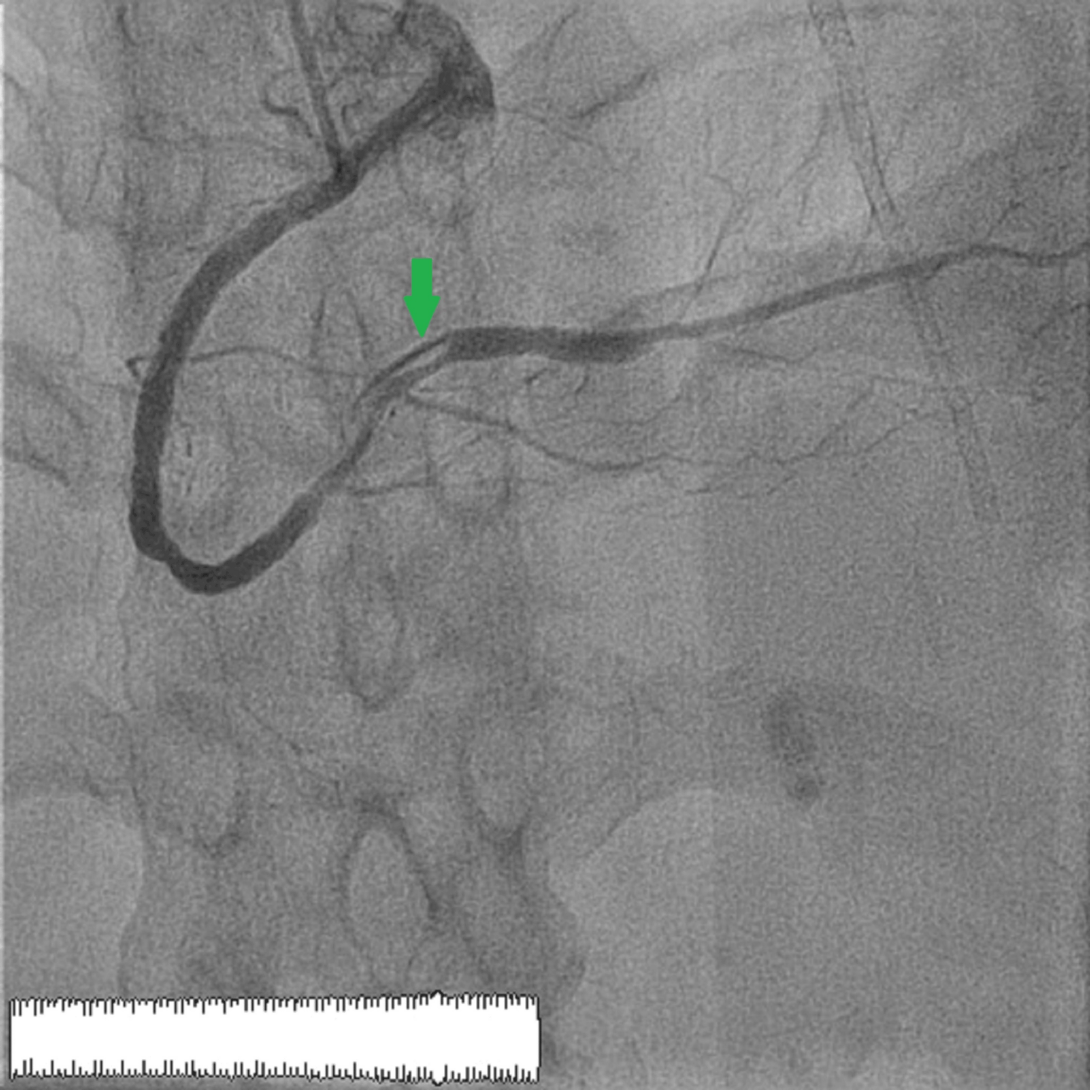
Cranial 30-degree view showing the reestablishment of flow with the persistence of a slight thrombus distal to the right coronary artery and the proximal part of the posterior retro-ventricular artery. The green arrow shows the migration of the thrombus toward the distal right coronary artery.

Immediate monitoring did not show any improvement in flow. The patient was admitted to the intensive care unit with close monitoring for hemorrhagic and ischemic signs. An angiographic follow-up after 48 hours demonstrated the resumption of TIMI III flow despite the migration of the thrombus toward the distal right coronary artery (Figures 4, 5). The patient returned to sinus rhythm 48 hours after intracoronary thrombolysis.

The patient was monitored for 48 hours and then discharged under medical treatment with a check-up at one month, demonstrating the maintenance of sinus rhythm with clinical and electrical improvement.

## Discussion

Fibrinolytic agents remain an effective means for the treatment of acute coronary syndromes with ST-segment elevation [3]. These agents are based on the activation of plasminogen, leading to fibrin degradation and the dissolution of thrombi, allowing the restoration of TIMI III flow in the coronary arteries. Several families of thrombolytic agents are available and are classified according to their affinity for fibrin, ranging from agents with low affinity, such as streptokinase, with moderate affinity, such as prourokinase, or with high affinity, such as tenecteplase [4].

The association of angioplasty with fibrinolytic therapy gives better results in terms of improvement of coronary flow; however, it increases the risk of hemorrhage [5]. In fact, in the French FAST-MI register, the early use of intravenous thrombolysis followed by a strategy of extensive angioplasty resulted in survival comparable to that of primary angioplasty [6]. Fibrinolytic agents are generally administered intravenously to treat acute coronary syndrome with ST-segment elevation within the first 12 hours. However, what about intracoronary thrombolysis?

The first study regarding the injection of intracoronary fibrinolytic agents was conducted by Sezer et al. in 41 patients with STEMI who were undergoing primary angioplasty with random assignment of 250 kU of intracoronary streptokinase. The results showed that low-dose intracoronary streptokinase immediately after primary PCI improved myocardial reperfusion but not long-term left ventricular size or function [7]. This study paved the way for further studies with higher-affinity agents such as tenecteplase. Indeed, the ICE-T-TIMI-49 study evaluated the effect of intracoronary tenecteplase in 40 patients undergoing primary angioplasty. This study showed that there was no difference in TIMI major bleeds, and the treatment with low-dose intracoronary tenecteplase appeared to be safe and well tolerated during Primary PCI. Intracoronary tenecteplase did not improve percent stenosis, but it reduced thrombus burden [8].

A meta-analysis by Rehan et al., including 12 randomized studies focusing on the use of intracoronary fibrinolytics, demonstrated that the use of these agents in association with PCI improves the clinical outcome as well as the microcirculation with no increase in hemorrhagic events [4].

In a new 2024 meta-analysis by Sahami et al., eight randomized clinical trials were included with a total of 1,208 patients demonstrating that adjunctive low-dose intracoronary thrombolysis during primary angioplasty is safe, associated with a trend toward lower major adverse cardiac events, and may improve surrogate markers of microvascular function [9].

These results showing the interest of fibrinolytic agents in improving microcirculation also suggest their interest in the treatment of the no-reflow phenomenon, whose pathophysiology consists mainly of a microvascular obstruction, incomplete dilatation of the lesion, coronary spasm, or dissection with or without in situ thrombosis [10].

In fact, it is possible that thrombi of different morphologies cause different types of microvascular obstruction at the time of primary PCI in patients with STEMI and probably require complementary therapeutic strategies [11]; hence, the interest of a low dose of intracoronary thrombolytic to dissolve the thrombus and improve microcirculation and reestablish the flow. The intracoronary administration of these agents allows a better concentration of the active agent in situ and a better dissolution of the thrombus.

In an Indian study of 1,020 patients who had undergone coronary evaluation, 380 underwent coronary angioplasty, with 32 patients developing a no-reflow state [10]. After intracoronary injection of tenecteplase, TIMI III flow was achieved in 91.67% patients, and TIMI II flow in 8.33% of patients [10]. However, a 2019 study by McCartney et al. among 440 patients randomized to receive alteplase 20 mg, alteplase 10 mg, or placebo showed that alteplase did not reduce microvascular obstruction, as assessed by MRI.

In a study by Datta among 580 patients, 44 cases of no-reflow benefited from a dose of 20 mg of intracoronary tenecteplase. TIMI II flow was reestablished in 32 patients after intracoronary tenecteplase (72%). Among the 12 failure cases, LAD artery involvement was most common in eight cases. The right coronary artery was involved in four patients. The one-month mortality rate in the no-flow group was 50%, and 6.25% in the successful recanalization group. The one-year mortality was 12.5% in the successful recanalization group and 66% in the no-flow group [12].

According to the 2023 European Society of Cardiology guidelines and the 2025 American College of Cardiology/American Heart Association guidelines, intracoronary thrombolysis is not part of standard reperfusion strategies in acute coronary syndromes and is not routinely recommended. Its use remains limited to selected bailout situations during PCI, with insufficient evidence to support routine use.

The occurrence of a no-reflow phenomenon is always possible in the angiography room. Its management is based on a set of means such as adenosine, nitroprusside, nicardipine, adrenaline, or verapamil [10]. However, the use of intracoronary fibrinolysis remains an interesting alternative, particularly when the thrombotic load appears significant, despite the fact that in the various randomized studies in the literature, there was no fixed dose of thrombolytic agents, but it depends on the molecule and the choices of the research team.

## Conclusions

The management of the no-reflow phenomenon remains difficult, given the varied pathophysiology of this phenomenon and the therapeutic arsenal available for its management. However, intracoronary thrombolysis for the management of no-reflow remains an attractive and interesting alternative with results that remain acceptable, but which need to be approved by studies on larger samples with more details on the dose and the administration methodology.
